# Infant Crying, Sleeping, and Feeding Problems in Times of Societal Crises: The Mediating Role of Parenting Stress on Parenting Behavior in Fathers and Mothers

**DOI:** 10.3390/children11121540

**Published:** 2024-12-19

**Authors:** Katharina Richter, Anna Friedmann, Volker Mall, Michaela Augustin

**Affiliations:** 1 School of Medicine and Health, Social Pediatrics, Technical University of Munich, 81675 Munich, Germany; katharina.richter@tum.de (K.R.); volker.mall@tum.de (V.M.); michaela.augustin@tum.de (M.A.); 2 German Center for Child and Adolescent Health (DZKJ), Partner Site Munich, 80337 Munich, Germany; 3 kbo-Kinderzentrum, 81377 Munich, Germany

**Keywords:** infant mental health, stress, sensitivity, responsivity, over-reactivity, pandemic, regulatory problems

## Abstract

Background/Objectives: Infant regulatory problems (RPs), i.e., crying, sleeping, and feeding problems, are associated with unfavorable outcomes in later childhood. RPs increased during the pandemic; however, their occurrence in the face of today’s societal challenges remains unclear. RPs are strongly linked to parenting stress and less positive parenting behaviors, but their interplay is less investigated. Methods: In this cross-sectional, questionnaire-based study (ntotal = 7039), we compared the incidences of crying, sleeping, and feeding problems in infants (0–2 years) in pandemic (npandemic = 1391) versus post-pandemic (npost-pandemic = 5648) samples in Germany. We also investigated the relationship between post-pandemic infant RPs and parenting behaviors with parenting stress as a potential mediator for fathers and mothers. Results: Crying/whining/sleeping problems (34.8%) and excessive crying (6.3%) were significantly more prevalent in the post-pandemic sample. In both mothers and fathers, infant RPs were significantly associated with less positive parenting behaviors. Parenting stress partially mediated this relationship. Conclusions: RPs in the post-pandemic era are even more prevalent than during the pandemic, highlighting the imperative for health care professionals to focus on infant mental health. Parenting stress emerges as an entry point for addressing the cycle of infant RPs and maladaptive behaviors in both fathers and mothers.

## 1. Introduction

Over the past few decades, longitudinal research has consistently demonstrated the harmful effects of early psychosocial stress on children’s mental health, with repercussions that can persist throughout the lifespan [1,2]. Psychosocial stress in families can arise from a variety of sources, including challenging living conditions (e.g., low socioeconomic status, lack of social support, or isolation), strained family relationships (e.g., between parents and children), and specific family burdens (e.g., health issues or limited childcare resources) [3]. The COVID-19 pandemic exacerbated these stressors, with families facing a significant increase in psychosocial pressure [4,5].

Concerns regarding child mental health emerged early in the pandemic, with national and international studies showing a marked rise in psychological problems among children and adolescents compared to pre-pandemic levels [6,7]. Very young children are known to be particularly vulnerable to the harmful effects of environmental stress [8], as they are almost entirely reliant on their parents for emotional and physical support during a critical phase of rapid brain development [9,10] in which key milestones in emotional regulation and learning have to be met [11]. When infants struggle to adapt to new conditions, it can manifest in regulatory problems, i.e., crying, sleeping, and feeding problems [12]. Their prevalence varies significantly depending on the diagnostic criteria and assessment tools used, but studies suggest that between 3% and 40% of physically healthy children experience these behavioral difficulties during infancy and early childhood [13,14,15]. Although regulatory problems may resolve within a few months, they can persist in the form of various behavioral irregularities into the preschool and elementary school years [16,17]. The persistence of regulatory issues increases the risk for social–emotional and cognitive difficulties. Children with multiple regulatory problems are particularly at risk for both internalizing and externalizing disorders later in life [18]. Hence, addressing these problems as early as possible is crucial.

The available studies investigating infant regulatory problems in the context of the pandemic predominantly suggest at least partially elevated levels of these difficulties compared to pre-pandemic periods [19,20]. Findings indicate that maternal pre- and postnatal concerns related to the pandemic, alongside anxiety and reduced wellbeing resulting from pandemic-related restrictions, may be associated with increases in infant regulatory problems [21,22,23,24,25]. The CoronabaBY study, which examined psychosocial stress factors such as parenting stress and mental health problems in families with children aged 0–3 years in Southern Germany [25], revealed an increase in infant mental health problems during the course of the pandemic. Surprisingly, infant crying, sleeping, and feeding problems did not follow a linear pattern in response to societal restrictions but remained at a distinctively high level compared to pre-pandemic data even after the reduction in pandemic restrictions. The findings suggest a staggered negative impact of pandemic-related factors on young children’s mental health that may still be present today, even if the pandemic is fading from public attention. This influence most likely operates indirectly through the wellbeing and mental health of the parents, which, in turn, shapes the emotional and psychological development of their infants. There is evidence from the pandemic period that psychological distress grows with the number of stressors present [26]. Since the aftermath of the pandemic, new stressors like, e.g., inflation, the climate crisis, and the ongoing wars in Ukraine and the Middle East, have emerged and, hence, families have had no time to rest navigating from one societal crisis to the next [27,28,29]. While societal crises might differ as to how they impact families (e.g., the pandemic having possibly combined direct and indirect effects and the recent wars probably having a more indirect influence in countries not involved in the conflict), it is evident that they occur as stress factors for families. This might be particularly critical in families with young children, as the transition to parenthood is already a challenging process. Family roles are often not yet established, and routines are not yet in place [30]. This may lead to an accumulation of family-related and societal stress factors in early parenthood with potential extensive impacts on infant mental health. Parents with their young children have indeed been identified as a particularly vulnerable group for mental health risks in this context. However, up to date, it remains largely unclear whether infant mental health problems in the post-pandemic period are as pronounced as they were during COVD-19.

Regulatory problems are usually demanding for parents, often leading to frustration, feelings of overwhelm, and negative emotions towards both the child and the parental role, which can strain the parent–child relationship [12]. Parenting stress and non-functional parenting behavior might further reinforce infant crying, sleeping, and feeding problems and could therefore be the relevant counseling entry points to break the cycle of negative family dynamics [31].

A shaping factor for the parent–child relationship is parenting behavior. Positive parenting behaviors, i.e., parental sensitivity and responsiveness, characterized by timely and appropriate responses to the child’s emotional and physical cues, are crucial for child mental health, particularly in early childhood [32,33]. Sensitive and responsive parenting behaviors promote positive interactions with the child, which, in turn, encourage positive emotions and cognitions about the parenting role [32,34]. However, in the context of infant regulatory problems, a parent’s ability to maintain this sensitivity and responsiveness can be impaired, making it harder to perceive and address the child’s needs effectively [11]. Parents of children with crying, sleeping, or feeding problems may become more insecure towards their children’s emotional signals, leading to more inadequate parenting behaviors, such as over-reactivity (exaggerated emotional responses or impulsive reactions to a child’s behavior) [35].

One key factor associated with less positive parenting behaviors is parenting stress, i.e., the burden resulting from the demands of the parenting role [36]. As caring for children with crying, sleeping, and feeding problems is especially demanding, parenting stress is also known to be particularly pronounced in families encountering these infant mental health issues [37,38].

Taken together, regulatory problems are known to involve a complex interplay of infant behavioral problems in at least one developmental area, pronounced parenting stress, and dysfunctional interaction patterns when dealing with the infant’s behavior [11,12]. Pandemic-based research has shown that parent–child relationships suffered [39], less positive parenting behaviors emerged [40], and parenting stress increased steadily over the course of COVID-19 [41]. First insights even showed a further increase in parenting stress following the declared end of the pandemic [42]. These results seem highly relevant in the context of regulatory problems today; however, their interplay is not yet clear. To the best of our knowledge, few studies have examined these variables within one integrated model, and research on infants with regulatory problems—such as problems with crying, sleeping, and feeding—remains notably limited in this context. Given the practical and clinical significance of these early regulatory difficulties, it is advisable for future research to place greater emphasis on infant crying, sleeping, and feeding problems within such models.

The existing literature on infant mental health is overwhelmingly based on mothers as caregivers. However, a broader societal trend has been observed over time, with an increasing expectation and willingness among fathers to take on a more active role in childrearing [43], reflected also by the rising number of fathers taking parental leave [44]. This shift has particularly intensified since the pandemic; due to working from home and limited access to childcare, fathers spent more time with their children and became more actively involved in daily family life and its challenges [45,46,47]. Some of these changes, such as remote working, have persisted post-pandemic. However, as most mothers still bear the majority of the caregiving responsibilities [48], fathers have often been overlooked in the research up to this point, negating their evolving roles in the family system.

The aim of this research is to deepen our understanding of the occurrence of infant crying, sleeping, and feeding problems in the post-pandemic context, as well as the dynamic with parenting stress and parenting behavior considering not only mothers but also the so-far-understudied group of fathers of infants with mental health problems. A thorough understanding will enable the development of more effective support strategies tailored to the specific needs of families, ensuring that both children and parents receive the appropriate care and intervention to promote healthy development and wellbeing also in times of societal crises.

Against this background, we aimed to answer the following research questions:

Are infant mental health problems in the so-called ’post-pandemic era’ as pronounced as they were during the pandemic? To answer the first research question, we cross-sectionally compared the incidences of crying, sleeping, and feeding problems in a sample of families with children aged 0–2 years old collected during the pandemic with a sample surveyed after the pandemic.

Are infant crying, sleeping, and feeding problems associated with fathers’ and mothers’ parenting behaviors, more precisely, their sensitivity, responsivity, and over-reactivity? Does parenting stress mediate this relationship? To answer the second research question, we investigated fathers and mothers in the post-pandemic group.

## 2. Materials and Methods

This study is a cross-sectional comparison between a sample of parents with infants between 0 and 2 years surveyed during the pandemic and a sample investigated post-pandemic. We drew the samples from two datasets: a total of 2940 parents participated in the CoronabaBY study conducted between February 2021 and March 2022 in Bavaria (Southern Germany). Of these, 1391 (47.31%) were mothers and fathers of infants aged 0 to 2 years, who formed the pandemic sample for the present investigation. For further details on the CoronabaBY study, see [25,41] or [49]. The post-pandemic sample was drawn from the JuFaBY (Junge Familien in Bayern: Young Families in Bavaria) study, which is an ongoing investigation of psychosocial stress in families with young children (up to primary school age) in Bavaria. In this project, 20,807 caregivers participated between February 2023 and August 2024, of whom 5648 (27.14%) were parents of infants between 0 and 2 years old and thus were part of the post-pandemic sample. The study sample comprised a total of 7039 participants. Study protocols for both the CoronabaBY and JuFaBY projects were approved by the Ethics Committee of the Technical University of Munich (CoronabaBY: vote no. 322/20 S; JuFaBY: vote no. 2022-483_1-S-KH).

### 2.1. Participants and Procedure

All participants were recruited through the smartphone app ‘My Pediatric Practice’ (www.monks-aerzte-im-netz.de) (accessed on 10 November 2024) and were surveyed using a digital questionnaire. The app is a widely used tool in pediatric practice aimed at facilitating communication between pediatricians and parents in outpatient pediatric care. An invitation to participate in this study was sent to all app users with children in the relevant age group. Parents were informed about the project via a detailed information text and could digitally consent to participate if interested. The invitations to participate in the survey were linked to the regular check-ups conducted as part of pediatric preventive care. These check-ups occur in Germany at the ages of three weeks, three months, six months, twelve months, and then annually until school entry. About 97% of parents participate in these examinations [50]. Only parents who could not understand the project information due to a lack of German language skills were excluded from participation. After giving informed consent, the app-using parent filled out digital questionnaires. As an incentive and a token of appreciation, families received a small gift for their children. For further details, refer to publications such as [25,41].

Data collection was based on standardized questionnaires, which were presented online in the ‘My Pediatric Practice’ app and could be completed and submitted by participating parents. In addition to infant crying, sleeping, and feeding problems, parenting stress, parenting behavior (sensitivity, responsivity, over-reactivity), as well as sociodemographic characteristics were recorded.

### 2.2. Measurements

#### 2.2.1. Sociodemographic Characteristics

General demographic characteristics of the parents and their children were surveyed, including sex and age, the caregiving situation (single parent/no single parent), information on nationality and highest educational attainment, and the number of children in the household.

#### 2.2.2. Infant Crying, Sleeping, and Feeding Problems

On the ‘Questionnaire for Crying, Sleeping, and Feeding’ [51], the two subscales ‘Crying/Whining/Sleeping’ and ‘Feeding’, as well as the scale for ‘Overall Infant Regulatory Problems’, were used. Parents answered 38 questions about their infants’ behaviors. The answers were based on four-point Likert scales ranging from ‘never’ to ‘always’. Based on validated cut-off values, dichotomous outcomes for the areas ‘Crying/Whining/Sleeping’ (cut-off: 1.84; sensitivity: 87%; specificity: 92%) and ‘Feeding’ (cut-off: 1.27; sensitivity: 57%; specificity: 77%) were calculated as ‘problematic’ or ‘non-problematic’. Furthermore, a total score for ‘Infant Overall Regulatory Problems’ was recorded based on raw values. The questionnaire can also provide indications of excessive crying according to the Wessel criterion (‘Rule of Threes’: crying for three or more hours a day, on more than three days a week, for more than three weeks) [52]. The validity of the questionnaire was confirmed by the high internal consistencies of the scales and correlations with behavioral diaries and clinical diagnoses [51].

#### 2.2.3. Parenting Stress

To assess parenting stress, the parent domain of the German adaptation of the ‘Parenting Stress Index (PSI)’ (‘Eltern-Belastungs-Inventar’ (EBI)) [53] was used. High scores indicate limited parental resources for upbringing and caring for the child. The parent domain includes the following subscales: ‘health’ (parental health impairment as a cause or result of parenting stress), ‘isolation’ (lacking integration in social networks), ‘role restriction’ (perceived limitations as a result of being a parent), ‘parental competence’ (parental doubt about their own ability to manage upbringing and care for their child), ‘attachment’ (emotional relation of the parent to the child), ‘depression’ (worries and negative thoughts due to the parental role), and ‘spouse-related stress’ (as a result of being a parent). Answers were given on a 5-point Likert scale ranging from 1 = strongly agree to 5 = strongly disagree, resulting in a possible score range of 28–140, which can be converted into EBI Total Score T-values. The three cut-off categories for each subscale and the whole parent domain were ‘not stressed’ (T-value < 60), ‘stressed’ (T-value = 60–69), and ‘strongly stressed’ (T-value ≥ 70). The internal consistency of the parent domain was proven to be good (α = 0.93), and the retest reliability after one year was shown to be r = 0.87. Correlations with stress indicators and related constructs resulted in the assumption of the test validity [54].

#### 2.2.4. Parenting Behavior

The ‘Comprehensive Early Childhood Parenting Questionnaire (CECPAQ)’ assesses parental behavior in early and middle childhood [55], drawing on attachment theory and social learning theories. The original questionnaire consists of 54 items and encompasses five core dimensions of parental behavior: ‘parental support’, ‘stimulation’, ‘structuring’, ‘positive discipline’, and ‘harsh discipline’. Each dimension is further divided into two to three subscales, allowing for analysis at both the dimension and subscale levels. The study focused on three selected subscales based on suitability for the age group of 0–2 year olds. The subscales for ‘responsiveness’ and ‘sensitivity’ are part of the ‘parental support’ dimension, while the subscale for parental ‘over-reactivity’ belongs to the ‘structuring’ dimension. Responses to the questionnaire are measured on a six-point scale, ranging from ‘never’ to ‘always’. Cronbach’s alpha of 0.75 for mothers and 0.77 for fathers in the structuring scale, and 0.88 for both parents in the support scale were shown to be good/acceptable. Significant correlations with parenting stress and child behavioral problems indicate the high validity of the questionnaire [55].

#### 2.2.5. Statistical Analysis

The data collected were initially provided to the research team by the operators of the ‘My Pediatric Practice’ app in the form of an Excel file for further processing. The datasets were then imported into the statistical software IBM SPSS Statistics Data Editor 28.0 for Windows. Since participants were required to submit fully completed questionnaires, there were very few missing data points. Individual missing data were excluded from the analysis.

To address the first research question, we tested whether infant crying, sleeping, and feeding problems differed between the post-pandemic group (*n* = 5648) and the pandemic group (*n* = 1391). Sociodemographic characteristics of the pandemic (CoronabaBY) and post-pandemic (JuFaBY) samples were analyzed for potential differences using Chi-square or Welch tests. To examine potential differences in the presence of problems (‘problematic’ vs. ‘not problematic’ according to the cut-off) in terms of crying and sleeping (crying/whining/sleeping subscale, SFS), feeding (feeding subscale, SFS), as well as excessive crying according to the ‘Rule of Threes’ (excessive crying, SFS) between the pandemic and post-pandemic groups, Chi-square tests were conducted. In case the cell frequencies were below 5, the Fisher Exact Test was calculated. Additionally, to assess the strength of the association between the variables, the effect size Phi (φ) was evaluated.

For the second research question, mothers and fathers in the post-pandemic sample were analyzed for possible differences in their demographic characteristics using the Chi-square or Welch test. We conducted three mediation models for each father and mother to investigate the relationship between overall infant regulatory problems (Infant Overall Regulatory Problems, SFS) and paternal and maternal parenting behavior (model 1: sensitivity subscale, CEQPAQ; model 2: responsiveness subscale, CEQPAQ; and model 3: over-reactivity subscale, CEQPAQ) and whether this relationship was mediated by paternal or maternal parenting stress (EBI Total Score T-values). The mediation analyses were performed using Hayes’ free PROCESS tool. Potential heteroscedasticity in the data was controlled for using heteroscedasticity-consistent standard errors (HC3). This method accounts for the possible non-constancy of the error variance, improving the accuracy of standard error estimates. The mediation analysis included the calculation of the direct effects for the individual paths and the indirect mediation effect at a 5% significance level. In cases of full mediation, the total effect loses significance. If the significance of the relationship between the independent and dependent variables remains after the inclusion of the mediator but is reduced, partial mediation can be assumed. The mediation effect was evaluated using the bootstrap method with 5000 resampling iterations, and confidence intervals for the indirect effect were calculated.

Finally, a post hoc power analysis using G*Power (https://www.psychologie.hhu.de/arbeitsgruppen/allgemeine-psychologie-und-arbeitspsychologie/gpower (accessed on 4 September 2024)) was conducted to determine whether the power 1 − *β* was within an acceptable range for small, medium, and large effects.

## 3. Results

### 3.1. Descriptives

The comparison between the samples showed that the parents in the post-pandemic (JuFaBY) group were, on average, younger than those in the pandemic group (CoronabaBY). Additionally, the post-pandemic group had a lower percentage of highly educated parents. The infant age was also, on average, lower in the post-pandemic group compared to that in the pandemic group. Due to differences in how nationality was recorded, the groups could not be compared on this variable. The effect sizes were consistently very small to small (see Table 1).

Comparing the mothers and fathers of the post-pandemic group for the second research question, we found that the fathers were, on average, older than the mothers. Additionally, a slightly higher proportion of mothers reported that one or both parents were born in Germany compared to fathers. The level of education also differed significantly, with a higher percentage of fathers having a higher education. There was also a notable difference in the single parent status, with mothers being more likely to be single parents. In terms of the children, the average age of the infants was lower among fathers than mothers, and a higher proportion of mothers reported having other children (siblings of the infant) (see Table 2). The effect sizes were very small to small except for the age difference in the parents (medium effect).

### 3.2. Infant Crying, Sleeping, and Feeding Problems During and After the Pandemic

According to the SFS, significantly more infants in the post-pandemic group showed problems in the areas of crying/whining/sleeping (*χ*^2^ (1, *n* = 7039) = 7.83, *p* = 0.005). This was also the case for excessive crying defined by the ‘Rule of Threes´ (*χ*^2^ (1, *n* = 7039) = 5.38, *p* = 0.020). The effect sizes were small. There were no significant differences between the two groups with regard to feeding problems. For detailed information, see Table 3.

### 3.3. Parenting Stress and Parenting Behavior in Mothers and Fathers in the Post-Pandemic Sample

The mean paternal parenting stress score in the parent domain of the EBI for this sample was 72.26 (*SD* = 19.30), and the mean maternal parenting stress score was 77.75 (*SD* = 20.34). The mean T-score was 55.42 (*SD* = 9.66) in fathers and 57.95 (*SD* = 9.61) in mothers, which, on average, fall within the ´not stressed´ range (<59). Additionally, the categorical classification based on the global stress score showed that 37.5% of fathers and 48.5% of mothers felt ‘stressed’ or ‘strongly stressed’ (see Table 4).

Fathers scored an average of *M* = 24.18 (*SD* = 3.80) (mothers: *M* = 26.17, *SD* = 3.18) on the responsiveness subscale and *M* = 18.58 (*SD* = 2.94) on the sensitivity subscale (mothers: *M* = 20.07, *SD* = 2.71). On the over-reactivity subscale, the average paternal score was *M* = 8.70 (*SD* = 3.36) (mothers: *M* = 9.75, *SD* = 3.77).

### 3.4. Relationships Between Infant Crying, Sleeping, and Feeding Problems, Parenting Stress, and Parenting Behaviors in the Post-Pandemic Sample

#### 3.4.1. Infant Crying, Sleeping, and Feeding Problems, Parenting Stress, and Sensitivity in Mothers and Fathers

**Figure 1:**
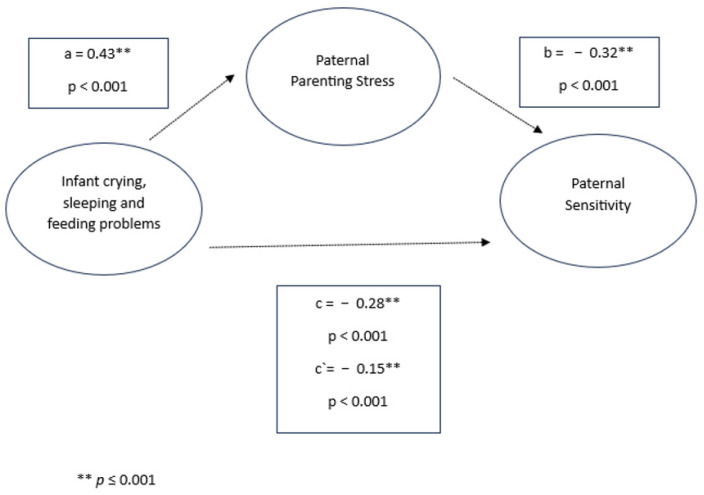
Infant crying, sleeping, and feeding problems, paternal parenting stress, and paternal sensitivity.

**Figure 2:**
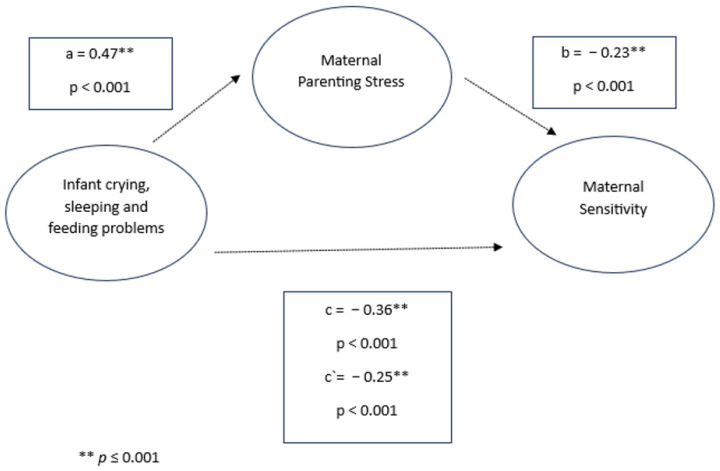
Infant crying, sleeping, and feeding problems, maternal parenting stress, and maternal sensitivity.

After parenting stress was included as a mediator in the model, infant crying, sleeping, and feeding problems were found to significantly negatively predict sensitive parenting behavior (mothers: *β* = −0.36, *p* < 0.001; fathers: *β* = −0.28, *p* < 0.001) and positively predict parenting stress (mothers: *β* = 0.47, *p* < 0.001; fathers: *β* = 0.43, *p* < 0.001). Moreover, parenting stress was significantly negatively associated with sensitivity in both parents (mothers: *β* = −0.23, *p* < 0.001; fathers: *β* = −0.32, *p* < 0.001). The indirect effects of both models (mothers: *β* = −0.11, 95% CI [−1.225, −0.946]; fathers: *β* = −0.14, 95% CI [−0.2005, −0.0864]) indicate a significant mediation [56]. Despite the mediated effect, the direct effect of infant crying, sleeping, and feeding problems on sensitivity remained significant (mothers: *β* = −0.25, *p* < 0.001; fathers: *β* = −0.15, *p* < 0.001), suggesting a partial mediation (see Figure 1 and Figure 2).

#### 3.4.2. Infant Crying, Sleeping, and Feeding Problems, Parenting Stress, and Responsiveness in Mothers and Fathers

**Figure 3:**
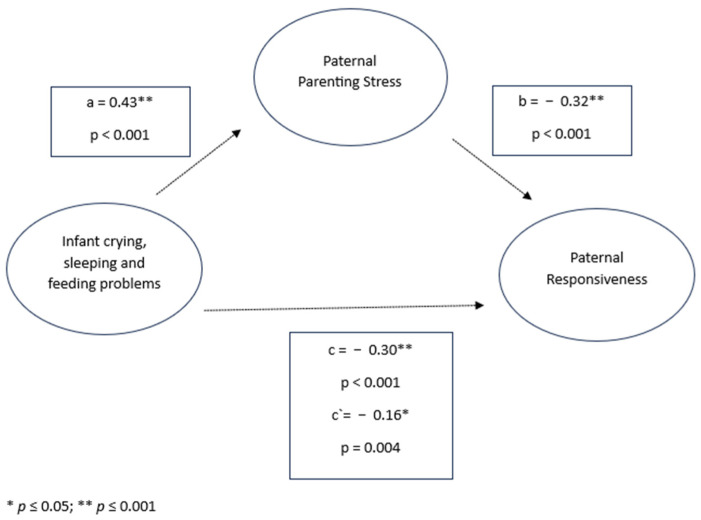
Infant crying, sleeping, and feeding problems, paternal parenting stress, and paternal responsiveness.

**Figure 4:**
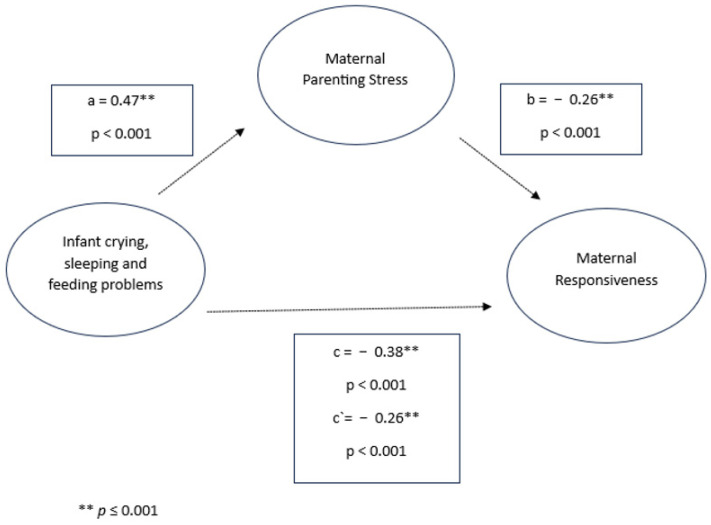
Infant crying, sleeping, and feeding problems, maternal parenting stress, and maternal responsiveness.

In the mediation models, infant crying, sleeping, and feeding problems were significantly associated with parenting stress and responsive parenting behavior in both mothers and fathers. Specifically, infant crying, sleeping, and feeding problems were positively associated with parenting stress (mothers: *β* = 0.47, *p* < 0.001; fathers: *β* = 0.43, *p* < 0.001) and negatively associated with responsive parenting behavior (mothers: *β* = −0.38, *p* < 0.001; fathers: *β* = −0.30, *p* < 0.001). Parenting stress was also negatively associated with responsiveness in both parents (mothers: *β* = −0.26, *p* < 0.001; fathers: *β* = −0.32, *p* < 0.001). Significant indirect effects were observed in both models, indicating a significant mediation (mothers: *β* = −0.12, 95% CI [−0.1353, −0.1076]; fathers: *β* = −0.14, 95% CI [−0.2068, −0.0837]). Despite the mediated effect, the direct effect of infant crying, sleeping, and feeding problems on responsiveness remained significant for both mothers (*β* = −0.26, *p* < 0.001) and fathers (*β* = −0.16, *p* = 0.004), indicating partial mediation (see Figure 3 and Figure 4).

#### 3.4.3. Infant Crying, Sleeping, and Feeding Problems, Parenting Stress, and Over-Reactivity in Mothers and Fathers

In the mediation model examining the effects of infant crying, sleeping, and feeding problems on parenting behaviors, parenting stress emerged as a significant mediator in the relationship between infant difficulties and over-reactive parenting for both mothers and fathers. Infant crying, sleeping, and feeding problems were positively associated with parenting stress (mothers: *β* = 0.47, *p* < 0.001; fathers: *β* = 0.43, *p* < 0.001), which, in turn, was associated with higher levels of over-reactive parenting behavior (mothers: *β* = 0.42, *p* < 0.001; fathers: β = 0.41, *p* < 0.001). Additionally, infant symptoms had a direct effect on over-reactivity, with significant values observed in both parents (mothers: *β* = 0.31, *p* < 0.001; fathers: *β* = 0.34, *p* < 0.001).

**Figure 5:**
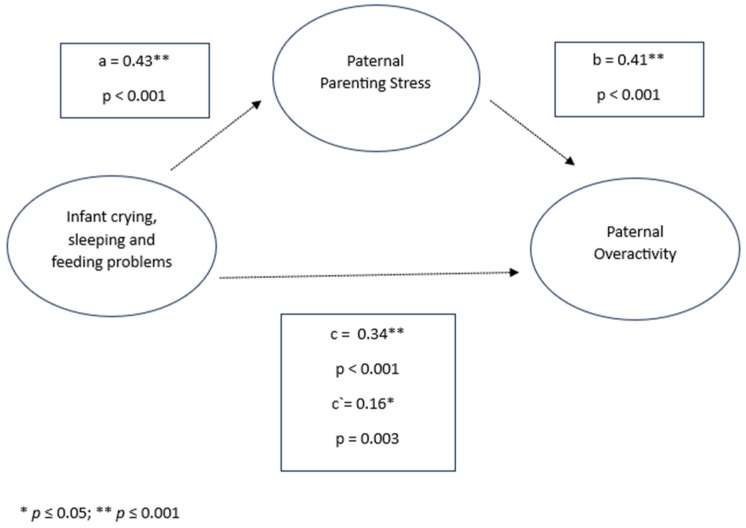
Infant crying, sleeping, and feeding problems, paternal parenting stress, and paternal over-reactivity.

**Figure 6:**
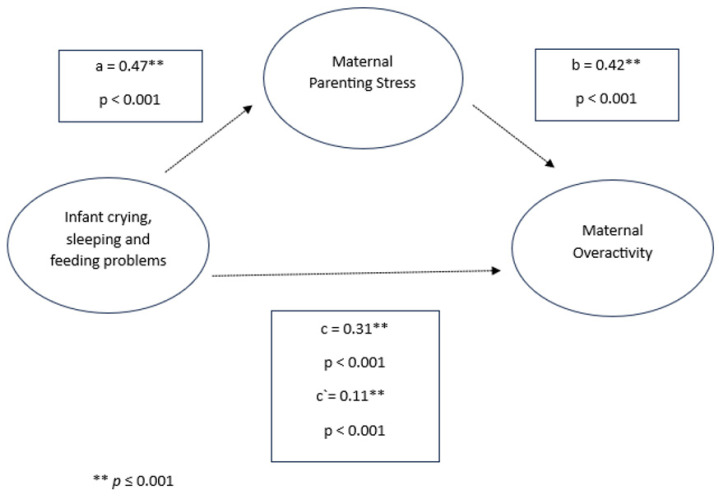
Infant crying, sleeping, and feeding problems, maternal parenting stress, and maternal over-reactivity.

The indirect effect of infant crying, sleeping, and feeding problems on over-reactive parenting behavior through parenting stress was significant for both mothers and fathers (mothers: *β* = 0.20, 95% CI [0.1847, 0.2150]; fathers: *β* = 0.18, 95% CI [0.1176, 0.2465]). Despite this mediated effect, the direct effect of infant crying, sleeping, and feeding problems on over-reactivity remained significant for both parents (mothers: *β* = 0.11, *p* < 0.001; fathers: *β* = 0.16, *p* = 0.003), suggesting that parenting stress partially mediated the relationship between infant difficulties and over-reactive parenting across both the maternal and paternal models (see Figure 5 and Figure 6).

## 4. Discussion

In a cross-sectional comparison study of pandemic (*n* = 1391) and post-pandemic (*n* = 5648) samples, including mothers and fathers with children aged 0–2 years, we examined whether the prevalence of infant crying, sleeping, and feeding problems differed between these two periods and whether parenting stress mediated the relationship between infant mental health problems and parenting behavior in the post-pandemic group. Our findings indicated significant differences in the prevalence of infant crying and sleeping problems as well as excessive crying between the pandemic and post-pandemic samples, suggesting that infant regulatory problems remain notably pronounced and are partially even more highly evident than during the pandemic. Maternal and paternal parenting stress partially mediated the association between infant crying, sleeping, and feeding problems and parenting behaviors with consistently small observed effects.

Examining our results in detail, the comparison with the pandemic reference sample revealed that significantly more crying/whining/sleeping problems and excessive crying were reported for infants of the post-pandemic group. Infant crying/whining/sleeping problems and excessive crying were reported by 34.8% and 6.3% of the parents in the post-pandemic sample, respectively. Feeding difficulties appeared in 35.1% of the infants in this group. Since regulatory problems are known to be transient in infancy and early childhood and vary significantly according to the age and developmental stage, classifications remain challenging. Yet, we found the rates to be relatively high in comparison to pre-pandemic reference studies in non-clinical samples, where the incidences for all these problems usually range up to around 20% [12,57,58,59,60,61]. The observed proportion of infants with regulatory problems also exceeded the levels found during the pandemic in prior studies, suggesting that infant mental health is particularly vulnerable in times of societal crises.

Theoretical explanations for our result include the fact that infants are particularly susceptible to environmental influences, with primary caregivers and the family environment serving as dominant influencing factors in their early development [62,63]. Current studies on parenting stress indicate that the parenting burden remains alarmingly high post-pandemic [42]. The prolonged period of anxiety and uncertainty, e.g., due to fear of infection, disrupted daily routines, and childcare closures, during the pandemic might have led to a state of mental exhaustion in parents [64]. Furthermore, the long-term effects of the crisis—despite the pandemic being considered over—may be compounding with the impacts of new societal challenges, such as the existential threat of climate change, economic inflation, and the wars in Ukraine and the Middle East. Today’s families are placed under unprecedented strain, which might lead to potential fatigue due to a cumulation of social challenges. Societal crises also represent a risk factor for parental anxiety and depression [42], which, in turn, are known to have potential negative effects on children’s mental health [65]. However, the influence of the present societal crises on infant mental health will have to be investigated in future studies since we cannot confirm these ideas on the basis of our data.

Our mediation models revealed that infant crying, sleeping, and feeding problems were linked to reduced maternal and paternal sensitivity and responsiveness along with increased over-reactive behaviors in both parents. Due to the lack of comparable studies with corresponding models, the individual paths can primarily be classified within the existing literature. Research has demonstrated that positive parenting behaviors can support and strengthen children’s regulatory capacities, suggesting that sensitive and responsive parental actions positively impact child regulation [66]. Conversely, evidence also indicates a bidirectional relationship, whereby child and parent behaviors mutually shape one another. For instance, regulatory problems in children may contribute to increased parental uncertainty, especially when conventional soothing methods prove ineffective, potentially diminishing parental confidence. This interplay can, in turn, influence parental behavior, which may inadvertently reinforce regulatory problems in the infant [67,68]. Since the design of this study does not allow for conclusions about the possible causal directions, further longitudinal studies should be conducted in this regard.

Additionally, infant crying, sleeping, and feeding difficulties positively predicted parenting stress in our study. In line with studies on parenting stress related to regulatory problems [38,69], a heightened infant symptomatology was associated with increased parenting stress. Furthermore, stressed parents demonstrated lower sensitivity and responsiveness, paired with more over-reactive parenting behaviors. These findings also add to the existing literature on parenting stress and behavior in parents, which has shown that higher stress levels correlate with harsher and more controlling parenting practices [40,70,71]. Parenting stress can also impair the ability to perceive and respond accurately to children’s needs [33,72]. In our study, maternal and paternal parenting stress partially mediated the link between infant crying, sleeping, and feeding problems and parenting behaviors. The models for mothers and fathers aligned in the direction of direct paths and demonstrated comparable effect sizes, with consistently small mediation effects in both cases, suggesting that these relationships may be similarly applicable for both parents. This implies that infant crying, sleeping, and feeding difficulties could partly contribute to increased parenting stress for both mothers and fathers, potentially reducing their parenting capacity by lowering their sensitivity and responsiveness while increasing their over-reactivity. While fathers still play a minor role in the research related to infant mental health, during the pandemic, noticeable changes emerged in family dynamics, including an increased involvement of fathers in the daily care and upbringing of their children. Fathers tend to spend more time in the everyday life of their children [45]. This makes it all the more important to bring fathers and their influence on the family system into greater focus in future research.

The interplay between infant regulatory problems, parenting stress, and parenting behavior is central to understanding the dynamics surrounding crying, sleeping, and feeding difficulties within the family system. The reciprocal nature of these challenges indicates that while infant difficulties can impact parental sensitivity and responsiveness, parenting behaviors, in turn, influence the course of these issues [66]. Recognizing this cycle provides valuable opportunities for intervention, potentially breaking the loop and lessening the impact of regulatory problems on both parents and infants, thereby increasing the chances of symptom relief. Given that parents often seek support only once regulatory issues are already present, we consider it particularly relevant in clinical practice to view already existing infant regulatory problems as a starting point for exploring the complex mechanisms within the family system.

Some strengths and limitations need to be considered when interpreting the underlying results. While the pandemic and post-pandemic samples were relatively similar in structure, they differed in terms of the parental age, infant age, presence of siblings, and educational background. We decided against controlling for these factors due to the very small to small effect sizes and also based on theoretical considerations: to the best of our knowledge, no studies specifically link parental age to infant regulatory problems. Although findings regarding the influence of having siblings/the birth order are somewhat inconsistent, most of the available data suggest no significant effects on infant crying or sleep patterns [73,74,75]. Evidence on the association between parental education and infant regulatory issues is very limited; to the best of our knowledge, there is one study that indicates that a lower educational background may be a risk factor for infant crying, sleeping, and feeding problems [76]. However, this study was from Turkey and this result cannot be fully transferred to the German context. In addition, the total sample consisted of mainly high educated parents, making it less likely to act as a confounding factor.

Differences also emerged with respect to the child’s age. Typically, the crying duration increases in the initial postnatal weeks, peaking around four to six weeks and then declining at around three to four months [77,78]. Since both samples, on average, were beyond this age range, age-related differences in crying behavior were not expected. Infant sleep patterns are variable and continually developing, but they generally start to stabilize at around five to six months, with longer nighttime sleep and increased sleep consolidation [79]. Both groups fell within this age range, reducing the likelihood of age-related effects.

The sample of mothers and fathers in the post-pandemic sample also differed significantly in terms of single parenthood, their educational level, and the presence of additional children. Yet, the inherent differences between mothers and fathers, especially with regard to being a single parent and education, are a reality that must be faithfully represented in the research. Accurately capturing these distinctions is crucial for producing findings that genuinely reflect the complexities of real-life dynamics. Overall, the sample primarily consisted of highly educated parents from a German background, resulting in a limited generalizability of the findings to other populations. It should also be considered that the mothers and fathers in the sample did not come from the same families; therefore, it remains unclear whether the perception of the child symptoms of one parent is influenced by the other parent.

Furthermore, the analysis is based on a cross-sectional design, which limits the ability to draw causal conclusions regarding the relationships between infant regulatory problems, parenting stress, and parenting behavior. Additionally, the small effect sizes observed in the mediation models suggest that the findings should not be overinterpreted. While they provide important initial insights, they do not yet establish a clear, clinically relevant effect. Nevertheless, this study represented an initial investigation of constructs that increase the understanding of infant regulatory problems as a triad of the child behavior, parenting burden, and parenting behavior within a single model and for both mothers and fathers of infants. As such, it provides valuable starting points and insights for further research in this area. The use of self-report measures for assessing infant symptoms and parenting behavior is another limitation, though standardized and validated instruments were employed.

Despite these limitations, the study presents several strengths. First, our study offers a comparison between distinct pandemic and post-pandemic groups with a considerably high sample size, providing valuable insights into infant mental health during and after the pandemic. Furthermore, this study is one of the first to examine the role of both mothers and fathers in the context of infant regulatory problems, particularly in light of the changes in family structures during the pandemic.

## 5. Conclusions

In conclusion, our findings underscore the continued significance of addressing infant mental health in the post-pandemic period. In light of the potentially lasting effects of the pandemic on parents and, subsequently, their young children, and the increasing influence of various societal challenges, it is imperative that both research and practice place greater emphasis on the wellbeing of infants and their families. Additionally, our results provide valuable insights into the complex interplay between infant regulatory problems, parenting stress, and parenting behaviors within the broader family system. A comprehensive understanding of these inter-related factors is, amongst others, crucial for developing effective support strategies aimed at enhancing the wellbeing of both the infant and the family as a whole. In particular, parenting stress—stemming from infant behavioral problems—emerges as a potential entry point for addressing the cycle of infant regulatory problems and maladaptive behaviors in both fathers and mothers. 

## Figures and Tables

**Table 1:** Demographic characteristics of the post-pandemic group and pandemic group.

	Post-Pandemic Group (JuFaBY) (*n* = 5648)		Pandemic Group (CoronabaBY) (*n* = 1391)		Statistical Significance (Effect Size)
Parents					
Parental age (years), mean (*SD*)	32.06 (4.55)			32.75 (4.57)	^b^ <0.001 * (0.15)
	%	*n*	%	*n*	
Fathers	5.6	314	6.8	95	^a^ 0.053 (−0.02)
(At least one) parent born in Germany	96.0	5423	91.0	1266	n.a.
Single parent	7.4	420	7.5	105	^a^ 0.864 (0.00)
Level of education					^a^ <0.001 * (0.06)
High	59.5	3362	62.4	847	
Low	38.6	2178	37.6	511	
Others	1.9	108	2.4	33	
Children					
Mean infant age in months, (*SD*)		4.86 (3.83)		5.47 (3.51)	^b^ <0.001 * (0.16)
	%	*n*	%	*n*	
Male	53.3	3013	51.8	720	^a^ 0.297 (0.02)
Siblings	30.5	1722	43.6	607	^a^ <0.001 * (−0.11)

^a^ Chi-square test (effect size Phi ϕ); ^b^ Welch test (effect size Cohen’s d); * *p* ≤ 0.05.

**Table 2:** Demographic characteristics of mothers and fathers in the post-pandemic sample.

	Mothers (*n* = 5334)		Fathers (*n* = 314)		Statistical Significance (Effect Size)
Parents					
Parental age (years), mean (*SD*)	31.91 (4.47)		34.50 (5.24)		^b^ <0.001 ** (−0.57)
	%	*n*	%	*n*	
Parent born in Germany	96.2	5129	93.6	294	^a^ <0.001 ** (0.10)
Predominantly single parent	7.8	417	1.0	3	^a^ <0.001 ** (−0.06)
Level of education					^a^ <0.001 ** (0.08)
High	58.6	3126	75.2	236	
Low	39.4	2103	23.9	75	
Others	2.0	105	1.0	3	
Children					
Infant age (months), mean (*SD*)		4.93 (3.85)		3.65 (3.37)	^b^ <0.001 ** (0.34)
	%	*n*	%	*n*	
Male	53.4	2851	51.6	162	^a^ 0.310 (0.02)
Siblings	31.2	1662	19.1	60	^a^ <0.001** (−0.06)

^a^ Chi-square test/Fisher Exact Test (effect size Phi ϕ); ^b^ Welch test (effect size Cohen’s d); ** *p* ≤ 0.001.

**Table 3:** Differences in infant crying, sleeping, and feeding problems in the post-pandemic and pandemic samples.

Infant Mental Health (CSF) (Above Cut-Off)	Post-Pandemic Group (JuFaBY) (*n* = 5648)		Pandemic Group (CoronabaBY) (*n* = 1391)		Statistical Significance (Effect Size ϕ)
Noticeable crying, feeding, and sleeping problems	%	*n*	%	*n*	
Excessive crying (Wessel criterion)	6.3	357	4.7	65	0.020 * (0.03)
Crying/whining/sleeping	34.8	1966	30.8	429	0.005 * (0.03)
Feeding	35.1	1981	36.7	510	0.273 (−0.01)

Chi-square test; * *p* ≤ 0.05.

**Table 4:** Parenting stress in mothers and fathers in the post-pandemic sample.

Parenting Stress (EBI)	%	*n*	%	*n*
Categorial Evaluation of the Parent Domain	Fathers (*n* = 314)		Mothers (*n* = 5334)	
No findings	62.4	196	51.5	2749
Stressed	29.9	94	34.5	1840
Strongly stressed	7.6	24	14.0	745

## Data Availability

The data necessary to reproduce the analyses presented here are not publicly accessible.
